# RETRACTION: Lyme Rashes Disease Classification Using Deep Feature Fusion Technique

**DOI:** 10.1111/srt.70291

**Published:** 2025-11-14

RETRACTION: G. Ali, M. Anwar, M. Nauman, M. Faheem, and J. Rashid, “Lyme Rashes Disease Classification Using Deep Feature Fusion Technique,” Skin Research and Technology 29, no. 11 (2023): e13519, https://doi.org/10.1111/srt.13519.

The above article, published online on 6 November 2023 in Wiley Online Library (wileyonlinelibrary.com), has been retracted by agreement between Skin Research and Technology; and John Wiley & Sons Ltd. The article was accepted for publication following an insufficient peer review process that does not meet the standards of the journal's peer review policy. Furthermore, the article includes several irrelevant citations, resulting in a background and rationale that lack adequate support from the literature. Finally, the article contains images sourced from the internet without permission or consent for publication. Accordingly, the article has been retracted. The authors have been informed of the retraction decision and disagree with it.

